# Exercise habits in adolescence and old age are positively associated with geriatric depressive symptoms: the Bunkyo Health Study

**DOI:** 10.3389/fpubh.2024.1405666

**Published:** 2024-11-19

**Authors:** Huicong Shi, Hiroki Tabata, Hikaru Otsuka, Hideyoshi Kaga, Yuki Someya, Abulaiti Abudurezake, Saori Kakehi, Hitoshi Naito, Naoaki Ito, Tsubasa Tajima, Yasuyo Yoshizawa, Ryuzo Kawamori, Hirotaka Watada, Yoshifumi Tamura

**Affiliations:** ^1^Department of Sports Medicine and Sportology, Graduate School of Medicine, Juntendo University, Tokyo, Japan; ^2^Sportology Center, Graduate School of Medicine, Juntendo University, Tokyo, Japan; ^3^Department of Metabolism and Endocrinology, Graduate School of Medicine, Juntendo University, Tokyo, Japan; ^4^Graduate School of Health and Sports Science, Juntendo University, Chiba, Japan; ^5^Juntendo Advanced Research Institute for Health Science, Tokyo, Japan; ^6^Faculty of International Liberal Arts, Juntendo University, Tokyo, Japan

**Keywords:** physical activity, teenage, older adults, GDS-15, geriatric depression, prevalence, sport

## Abstract

**Introduction:**

Exercise is a crucial method for preventing geriatric depression. In this cross-sectional study, we investigated the associations between exercise habits in adolescence and old age and geriatric depressive symptoms.

**Methods:**

This study used baseline data from the Bunkyo Health Study, a prospective observational cohort study investigating the preventive effects of physical activity on causative diseases requiring long-term care. This analysis included 1,629 older adults (687 men and 942 women) aged 65–84 years who participated in the Bunkyo Health Study. Participants were divided into four groups according to their exercise habits in adolescence and old age: never exercised (none-none; NN), exercised only in old age (none-active; NA), exercised only in adolescence (active-none; AN), and exercised in adolescence and old age (active-active; AA). Geriatric depressive symptoms were defined as the short version of the Geriatric Depression Scale score ≥ 5, including depression tendency. Multivariate-adjusted logistic regression models were used to estimate the odds ratios (ORs) and associated 95% confidence intervals in each group for the prevalence of geriatric depressive symptoms compared with the NN group.

**Results:**

The ORs for geriatric depressive symptoms were notably lower in the AN, NA, and AA groups than in the NN group in both older men and older women.

**Conclusion:**

These results indicate that older adults with exercise habits in adolescence and/or in old age exhibit a lower prevalence of geriatric depressive symptoms.

## 1 Introduction

Geriatric depression causes mood symptoms and decreases the quality of life (QOL) of older adults (1). Its prevalence ranges from 1 to 4% in older adults, with a higher prevalence in community-dwelling women (2). Although it is not sufficient to diagnose major depression, a previous study found that approximately 10–15% of older adults have clinical depressive symptoms (3). Geriatric depression is associated with suicidal ideation and a high rate of progression to dementia (4). Geriatric depression is associated with a higher severity of physical symptoms than depression in younger adults (5), and the presence of residual symptoms can increase recurrence rates (6). Thus, the prevention of geriatric depression is essential for enhancing the QOL of older adults.

Exercise is crucial for preventing depression in geriatric patients. Exercise in old age is well-established to prevent geriatric depression (7, 8). Additionally, exercise in adolescence may play a pivotal role in preventing geriatric depression. Regular adolescent exercise has been found to enhance cognitive function and promote neuroplasticity (9), potentially strengthening an individual’s resilience to depression later in life (10). Therefore, it can be inferred that a combination of exercise habits in both age periods (e.g., adolescence and old age) may have an additive effect in the prevention of geriatric depression, which remains to be seen.

Thus, in this study, we aimed to examine the associations between exercise habits in adolescence and old age and geriatric depressive symptoms. We hypothesized that exercise habits in both adolescence and old age could be effective in decreasing the prevalence of geriatric depressive symptoms.

## 2 Materials and methods

### 2.1 Study design and participants

This cross-sectional study used baseline data from the Bunkyo Health Study, a prospective observational cohort study investigating the preventive effects of physical activity on causative diseases requiring long-term care (11). The Bunkyo Health Study recruited older individuals aged 65–84 years living in Bunkyo-Ku, an urban area in Tokyo, Japan; 1,629 participants (687 men and 942 women) completed a two-day study examination at the Sportology Center between October 15, 2015, and October 1, 2018. Briefly, we evaluated cognitive function and performed a screening test for geriatric depressive symptoms using a questionnaire, assessed muscle strength and physical performance, performed brain lesion evaluations using magnetic resonance imaging, body composition and bone mineral density assessment using dual-energy X-ray absorptiometry, blood sample collection, and a 75-g oral glucose tolerance test (75-g oral glucose tolerance tests [OGTT]) after overnight fasting.

The study protocol was approved by the Ethics Committee of Juntendo University in September 2015 (first approval no. 2015061 and the latest revised version no. M15-0057-M08). The study was conducted in accordance with the principles of the Declaration of Helsinki. All participants were given a clear explanation of the study’s details. Following this, they provided written informed consent, indicating their voluntary agreement to participate.

### 2.2 Definition of geriatric depressive symptoms

The short version of the Geriatric Depression Scale (GDS-15) was used to assess depressive symptoms in older adults (12). GDS-15 scores range from 0 to 15, which can be divided to four groups: (1) Normal, 0–4 points; (2) Mild depressive symptoms, 5–8 points; (3) Moderate depressive symptoms, 9–11 points; (4) Severe depressive symptoms, 12–15 points. In this study, 5 or higher GDS-15 scores were defined as geriatric depressive symptoms, including depressive tendencies (13). The GDS-15 has been reported to be a reliable and valid screening tool for major depressive disorder across gender, ethnicity, and chronic illness status (13, 14).

### 2.3 Definition of exercise habits

Exercise habit data were collected by self-report using the Bunkyo Health Study baseline questionnaire. All participants were interviewed using the following questions: “Did you participate in sports club activities when you were in junior high school or high school?” and “Do you currently have exercise habits?” We then asked those who answered “yes” about their engagement in specific sports, club activities, current exercise types, and frequency. Additionally, current physical activity levels were evaluated using the International Physical Activity Questionnaire. We defined those who participated in sports club activities during junior high or high school as exercising in adolescence. For participants with only a junior high school education, their specific duration of exercise habits in adolescence was 3 years. However, for those with a high school or higher education, the duration may have been 3 or 6 years, reflecting exercise during junior high, high school, or both periods. Based on the definition of exercise habits in the National Health and Nutrition Exercise Survey in Japan, those who exercised more than twice a week for at least 30 min per session were defined as having exercise habits in old age. We also asked, “Did you have exercise habits at each age from your 20s to 50s?” Those who responded “yes” were defined as having exercise habits at each age range from their 20s to 50s.

### 2.4 Other measurements

In the upright position, height was measured within 0.1 cm using a stadiometer (YS-201-P; YAGAMI Inc., Nagoya, Japan). Body mass was measured by 0.1 kg using an electronic scale (InBody770; Biospace, Seoul, Korea). Body mass index (BMI) was calculated as body weight divided by height squared in meters. Self-administered questionnaires were used to determine age (years), years of education (years), smoking status (current or past) and living alone. Current or past smokers answered questions about the amount and years of smoking to calculate the Brinkman index: the number of cigarettes smoked per day multiplied by the number of years of smoking. Dietary intake was assessed using a Brief self-administered Diet History Questionnaire (BDHQ) to measure alcohol intake. The BDHQ has been validated in a previous study (15). Physical activity levels were evaluated using the International Physical Activity Questionnaire (IPAQ).

Medical history and medication information were recorded by a physician during the interviews using a semi-structured questionnaire. Hypertension was defined as systolic blood pressure ≥ 140 mmHg and/or diastolic blood pressure ≥ 90 mmHg or current use of antihypertensive medications. Blood samples were collected in the morning after an overnight fast to perform appropriate biochemical tests. Blood glucose and hemoglobin A1C levels were measured at the Commissioned Clinical Laboratory Center (SRL Inc., Tokyo, Japan). Diabetes mellitus was defined as fasting blood glucose ≥126 mg/dL and a 2-h blood glucose level ≥ 200 mg/dL after a 75-g oral glucose tolerance test, hemoglobin A1C ≥6.5%, or currently taking diabetes medication. Dyslipidemia was defined as low-density lipoprotein (LDL) cholesterol ≥140 mg/dL, high-density lipoprotein (HDL) cholesterol <40 mg/dL, triglycerides ≥150 mg/dL, or the current use of lipid-lowering agents. We combined hypertension, diabetes mellitus and dyslipidemia as cardiovascular risk factors. Serum Brain-derived neurotrophic factor levels (BDNF) were measured using a multiplex assay (MILLIPLEX MAP Human Myokine Magnetic Bead Panel; Merck, Darmstadt, Germany). Hippocampus volume (Vol) was assessed by Magnetic Resonance Imaging using a 0.3 T clinical MR scanner (AIRIS Vento, Hitachi, Tokyo, Japan). Hippocampus Vol was corrected by intracranial volume (ICV). Mild cognitive impairment was defined as a score ≤ 22 on the Japanese version of the Montreal Cognitive Assessment (MoCA-J).

### 2.5 Statistical analysis

All participants were divided into four groups according to their exercise habits in adolescence and old age as follows: (1) never exercised (none-none; NN), (2) exercised only in old age (none-active; NA), (3) exercised only in adolescence (active-none; AN), and (4) exercised in adolescence and old age (active-active; AA). Continuous variables were summarized by presenting the median and interquartile range of patients who contributed values. Categorical variables were summarized by presenting the frequency and proportion of patients in each category. Characteristics were compared using the Kruskal–Wallis and chi-squared tests for continuous and categorical variables, respectively. The NN group was used as the reference group, and logistic regression analysis was performed to estimate the odds ratio (ORs) and 95% confidence interval (CIs) for the prevalence of geriatric depressive symptoms in each group. Model 1 was adjusted for the following potential covariates: age (continuous variables), years of education (continuous variables), BMI (continuous variables), Brinkman index (continuous variables), and alcohol intake (continuous variables). Model 2 was adjusted for model 1 covariables plus diabetes mellitus (yes or no), cerebrovascular disease (yes or no), and mild cognitive impairment (yes or no). Model 3 was adjusted for model 2 covariables plus living alone (yes or no). Subsequently, multiple regression analysis was performed to determine the independent contribution of exercise habits in each age and potential covariates to the GDS-15 score; differences among the four groups were compared using analysis of covariance (ANCOVA), adjusted for the potential confounders as follows: age (continuous variable), years of education (continuous variables), BMI (continuous variable), Brinkman index (continuous variable), alcohol intake (continuous variables), former and current smoking status (yes or no), diabetes mellitus (yes or no), cerebrovascular disease (yes or no), mild cognitive impairment (yes or no) and living alone (yes or no). We adjusted for multiple comparisons using post-hoc Bonferroni correction. All statistical analyses were performed using IBM SPSS Statistics for Windows, version 28.0 (IBM Corp., Armonk, NY, USA). All tests were two-sided, with a 5% significance level.

## 3 Results

### 3.1 Demographic and baseline characteristics

The characteristics of the participants in the four groups, classified by the combination of exercise habits in adolescence and old age, are shown in Table 1. Among men, the participants were significantly younger in the AA group than in the NN group, and the BMI in the NA and AA groups was significantly lower than that in the NN group. The Brinkman indices of the AN and AA groups were significantly higher than those of the NN and NA groups. Alcohol intake was significantly higher in the AN and AA groups than in the NA group. The GDS-15 scores of the NA, AN, and AA groups were significantly lower than those of the NN group. Moreover, it was significantly lower in the AA group than in the AN group. Among participants with depressive symptoms, mild depressive symptoms were the most common, while severe depressive symptoms were the least frequent in both men and women. Among all categories of depressive symptoms, the NN group had the highest prevalence. The prevalence of geriatric depressive symptoms in men in the four groups was 29.3, 17.1, 17.8, and 15.6%, respectively. The prevalence of geriatric depressive symptoms was highest in the NN group. Among the women, age was comparable among the four groups. The Brinkman index and alcohol intake were significantly higher in the AA group than in the NN and NA groups. The GDS-15 scores in the NA and AA groups were significantly lower than those in the NN group, and the prevalence of geriatric depressive symptoms in women in the four groups was 28.4, 19.7, 19.1, and 19.5%, respectively. The prevalence of geriatric depressive symptoms was highest in the NN group.

**Table 1:** Participant characteristics.

	None-none	None-active	Active-none	Active-active	*p*-value
Men					
Number of subjects, *n* (%)	133 (19.4)	123 (17.9)	258 (37.6)	173 (25.2)	
Age, y	74 (69–79)	73 (69–78)	72 (68–77)	72 (68–75)^a^	0.007
Height, cm	164.8 (161.0–168.2)	165.7 (160.7–169.4)	166.0 (162.0–170.2)	166.4 (161.6–170.9)	0.087
Body mass, kg	66.0 (60.6–70.6)	63.3 (57.9–69.4)	65.9 (60.0–71.7)	65.0 (59.3–72.1)	0.134
Body mass index, kg/m^2^	24.2 (22.5–25.9)	23.5 (21.6–25.4)^a^	23.7 (22.1–25.5)	23.5 (22.0–25.6)^a^	0.043
Years of education, y	16 (12–16)	16 (14–16)	16 (12–16)	16 (16–16)^c^	0.010
Brinkman index	175 (0–650)	200 (0–800)	450 (23.8–845)^a, b^	500 (52.5–887.5)^a, b^	0.001
Past smoking status, *n* (%)	53 (60.2)^*^	117 (69.6)	121 (78.6)	210 (75.8)	0.008
Current smoking status, *n* (%)	12 (13.6)	24 (7.7)^*^	29 (18.8)^*^	37 (13.4)	0.035
Alcohol intake, g/d	11.6 (0.2–33.7)	5.4 (0–24.7)	17.1 (0.2–41.9)^b^	14.6 (1.1–43.2)^b^	0.008
Living alone, *n* (%)	13 (9.8)	12 (9.8)	39 (15.1)	20 (11.6)	0.318
Cardiovascular risk factors, *n* (%)	120 (90.2)	104 (84.6)	228 (88.4)	150 (86.7)	0.538
Hypertension, *n* (%)	104 (78.2)	90 (73.2)	195 (75.6)	114 (65.9)	0.067
Diabetes mellitus, *n* (%)	26 (19.5)	19 (15.4)	46 (17.8)	35 (20.2)	0.735
Dyslipidemia, *n* (%)	83 (62.4)	67 (54.5)	157 (60.9)	82 (52.6)	0.202
Cerebrovascular disease, *n* (%)	10 (7.5)	7 (5.7)	5 (1.9)^*^	12 (6.9)	0.038
Mild cognitive impairment, *n* (%)	34 (25.6)	37 (30.1)^*^	55 (21.3)	26 (15.0)^*^	0.014
Hippocampus Vol/ICV, %	0.39 (0.36–0.42)	0.40 (0.37–0.42)	0.39 (0.36–0.41)	0.39 (0.37–0.42)	0.184
Serum BDNF, pg/ml	19,259 (14822–21,528)	19,866 (16864–21,591)	18,690 (14491–21,486)	19,351 (15698–21,619)	0.378
Physical activity level (Mets hours/week)	0 (0–0)	24.5 (14.0–37.5)^a^	0 (0–2.7)^b^	24.5 (16.2–32.7)^a, c^	<0.001
GDS score	3 (1–5)	2 (1–3) ^a^	2 (1–4) ^a^	1 (0–3)^a, c^	<0.001
Geriatric depressive symptoms, *n* (%)	39 (29.3)^*^	21 (17.1)	46 (17.8)	27 (15.6)	0.013
GDS score classification, *n* (%)					
Normal (0 ~ 4 points)	94 (70.7)	102 (82.9)	212 (82.2)	146 (84.4)	0.031
Mild depressive symptoms (5 ~ 8 points)	29 (21.8)	18 (14.6)	39 (15.1)	25 (14.5)	
Moderate depressive symptoms (9 ~ 11 points)	8 (6.0)	2 (1.6)	7 (2.7)	2 (1.1)	
Severe depressive symptoms (12 ~ 15 points)	2 (1.5)	1 (0.8)	0	0		
Women					
Number of subjects, *n* (%)	303 (32.2)	198 (21.0)	267 (28.3)	174 (18.5)	
Age, y	73 (69–78)	74 (69–78)	72 (68–78)	72 (69–77)	0.335
Height, cm	152.3 (148.5–155.4)	152.1 (149.4–155.0)	153.0 (149.0–156.7)^a^	153.1 (149.5–157.6)^a, b^	0.014
Body mass, kg	52.3 (46.9–57.2)	51.4 (46.4–55.6)	52.4 (47.6–58.4)	52.8 (47.9–58.0)	0.185
Body mass index, kg/m^2^	22.6 (20.4–24.9)	22.2 (20.4–24.2)	22.7 (20.6–24.8)	22.4 (20.4–24.5)	0.608
Years of education, y	12 (12–14)	12 (12–15)	12 (12–14)	12 (12–14)	0.733
Brinkman index	0 (0–0)	0 (0–0)	0 (0–0)	0 (0–0)^a, b^	0.028
Past smoking status, *n* (%)	29 (17.5)	46 (13.7)	28 (18.9)	61 (20.8)	0.550
Current smoking status, *n* (%)	5 (3.0)	8 (2.4)	5 (3.4)	13 (4.4)	0.124
Alcohol intake, g/d	0.0 (0.0–1.7)	0.1 (0.0–2.5)	0.2 (0.0–3.5)	0.5 (0.0–8.5)^a, b^	0.020
Living alone, *n* (%)	75 (24.8)	76 (38.4)^*^	60 (22.5)^*^	47 (27.0)	0.001
Cardiovascular risk factors, *n* (%)	260 (85.8)	159 (80.3)	228 (85.4)	145 (83.3)	0.361
Hypertension, *n* (%)	201 (66.3)	111 (56.1)	163 (61.0)	99 (56.9)	0.076
Diabetes mellitus, *n* (%)	28 (9.2)	14 (7.1)	31 (11.6)	11 (6.3)	0.195
Dyslipidemia, *n* (%)	199 (65.7)	128 (64.6)	178 (66.7)	121 (69.5)	0.772
Cerebrovascular disease, *n* (%)	14 (4.6)	7 (3.5)	7 (2.6)	6 (3.4)	0.647
Mild cognitive impairment, *n* (%)	54 (17.8)	25 (12.6)	33 (12.4)	31 (17.8)	0.155
Hippocampus Vol/ICV, %	0.41 (0.39–0.44)	0.41 (0.38–0.44)	0.41 (0.39–0.43)	0.42 (0.39–0.44)	0.695
Serum BDNF, pg/ml	18,658 (13267–21,295)	19,226 (14479–21,421)	19,291 (14304–21,274)	19,106 (14278–21,717)	0.372
Physical activity level (Mets hours/week)	0.0 (0.0–4.6)	15.9 (8.0–24.5)^a^	0.0 (0.0–4.5)^b^	17.5 (9.6–25.0)^a, c^	<0.001
GDS score	3 (1–5)	2 (0.8–4) ^a^	2 (1–4)	2 (1–4) ^a^	0.001
Geriatric depressive symptoms, *n* (%)	86 (28.4)^*^	39 (19.7)	51 (19.1)	34 (19.5)	0.022
GDS score classification, *n* (%)					
Normal (0 ~ 4 points)	217 (71.6)	159 (80.3)	216 (80.9)	140 (80.5)	0.131
Mild depressive symptoms (5 ~ 8 points)	70 (23.1)	35 (17.7)	42 (15.7)	31 (17.8)	
Moderate depressive symptoms (9 ~ 11 points)	11 (3.6)	4 (2.0)	7 (2.6)	2 (1.1)	
Severe depressive symptoms (12 ~ 15 points)	5 (1.7)	0	2 (0.7)	1 (0.6)	

Values are shown as median (interquartile range) for continuous variables and frequencies (percentage) for categorical variables.

GDS, the Geriatric Depression Scale; Hippocampus Vol/ICV, hippocampus volume corrected by intracranial volume (ICV); BDNF, brain-derived neurotrophic factor levels; Mets, metabolic equivalent.

^aSignificant difference compared to the none-none group, bsignificant difference compared to the none-active group, csignificant difference compared to the active-none group.^

^*^p < 0.05, significant difference among groups according to the chi-square test.

### 3.2 Association between exercise habits among the four groups and the ORs of geriatric depressive symptoms

The ORs for geriatric depressive symptoms in the NA, AN, and AA groups compared with the NN group are shown in Table 2. In men, the ORs for geriatric depressive symptoms were significantly lower in the NA, AN, and AA groups than in the NN group after adjusting for model 3 (NA; OR: 0.48, 95% CI: 0.26–0.90; AN; OR: 0.51, 95%CI: 0.31–0.86; AA; OR: 0.45, 95%CI: 0.25–0.81). In women, the ORs for geriatric depressive symptoms were significantly lower in the NA, AN, and AA groups than in the NN group after adjusting for model 3 (NA; OR: 0.63, 95% CI: 0.40–0.97; AN; OR: 0.59, 95%CI: 0.40–0.89; AA; OR: 0.63, 95%CI: 0.40–0.99).

**Table 2:** Association between the combination of exercise habits and prevalence of geriatric depressive symptoms.

		Prevalence *n* (%)	Crude		Model 1^a^		Model 2^b^		Model 3^c^	
			OR (95%CI)	*p*-value	OR (95%CI)	*p*-value	OR (95%CI)	*p*-value	OR (95%CI)	*p*-value
Men	None-none (*N* = 133)	39 (29.3)	Ref.		Ref.		Ref.		Ref.	
	None-active (*N* = 123)	21 (17.1)	0.50 (0.27–0.90)	0.022	0.49 (0.27–0.91)	0.024	0.48 (0.26–0.89)	0.020	0.48 (0.26–0.90)	0.021
	Active-none (*N* = 258)	46 (17.8)	0.52 (0.32–0.85)	0.010	0.50 (0.30–0.84)	0.008	0.54 (0.32–0.90)	0.018	0.51 (0.31–0.86)	0.012
	Active-active (*N* = 173)	27 (15.6)	0.45 (0.26–0.78)	0.004	0.46 (0.26–0.83)	0.009	0.46 (0.26–0.83)	0.010	0.45 (0.25–0.81)	0.008
Women	None-none (*N* = 303)	86 (28.4)	Ref.		Ref.		Ref.		Ref.	
	None-active (*N* = 198)	39 (19.7)	0.62 (0.40–0.95)	0.029	0.62 (0.40–0.95)	0.029	0.63 (0.41–0.98)	0.040	0.63 (0.40–0.97)	0.036
	Active-none (*N* = 267)	51 (19.1)	0.60 (0.40–0.88)	0.010	0.58 (0.39–0.87)	0.008	0.60 (0.40–0.88)	0.010	0.59 (0.40–0.89)	0.011
	Active-active (*N* = 174)	34 (19.5)	0.61 (0.39–0.96)	0.033	0.63 (0.40–0.99)	0.044	0.63 (0.40–0.99)	0.046	0.63 (0.40–0.99)	0.046

^aModel l was adjusted for age, body mass index, years of education, alcohol intake, and Brinkman index.^

^bModel 2 was adjusted for Model 1 plus diabetes mellitus, cardiovascular diseases, and mild cognitive impairment.^

^cModel 3 was adjusted for Model 2 plus living alone.^

### 3.3 Comparison of the GDS-15 scores among four groups

**Figure 1:**
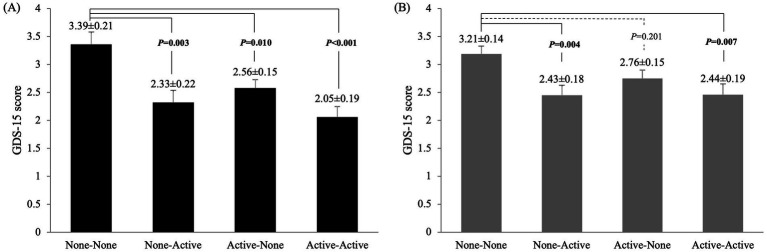
Comparison of GDS-15 scores among the four groups in (A) men and (B) women: no exercise in either period (none-none), exercise only in adolescence (active-none), exercise only in old age (none-active), and exercise in both periods (active-active). Values are presented as means ± SE. Adjusted variables: age, years of education, alcohol intake, Brinkman index, diabetes mellitus, cardiovascular disease, mild cognitive impairment and living alone.

Differences in the GDS-15 scores among the four groups were compared using ANCOVA after adjusting for potential confounders (Figure 1). In men, the GDS-15 scores were significantly higher in the NN group than in the other three groups (NA: *p* = 0.003; AN: *p* = 0.010; AA: *p* < 0.001). However, in women, the GDS-15 scores were significantly higher in the NN group than in the NA (*p* = 0.004) and AA groups (*p* = 0.007).

## 4 Discussion

In this study, we investigated the association between exercise habits in adolescence, old age, or both and the prevalence of geriatric depressive symptoms. The prevalence of geriatric depressive symptoms in the NA, AN, and AA groups was significantly lower than that in the NN group for both men and women.

We found that exercise habits in adolescence and old age were associated with a lower prevalence of geriatric depressive symptoms in both men and women. These results partially supported our hypothesis that exercise habits in both age groups might protect against geriatric depressive symptoms. Previous research has demonstrated that regular exercise in old age can benefit geriatric depression (16–18) and that regular exercise in adolescence is associated with a lower prevalence of depression in adolescence (19), which may affect the early onset of geriatric depression. Therefore, our findings are consistent with these results. Regular exercise can promote positive emotions and self-efficacy in adolescence (20, 21) and old age (22). Increased self-efficacy in individuals with exercise habits may promote greater well-being and reduce depressive symptoms later in life (23). However, our study did not find a synergistic effect of exercise habits on geriatric depressive symptoms in adolescence or old age. The reasons for this are unclear, and further research is required.

Conversely, the logistic regression analysis indicated an increased risk of geriatric depressive symptoms only in the NN group. We enquired about exercise habits from the 20s to the 50s and drew trajectories of exercise habits for depressed and non-depressed individuals (Supplementary Figure S1). We found that the proportion of people with exercise habits in the geriatric depressive symptoms group was lower than that in the non-geriatric depressive symptoms group at almost all ages, both for men and women. This diminished consistency in exercise habits over the lifespan mirrors the pattern observed in the NN group (Supplementary Figure S2), suggesting that this led to higher ORs for geriatric depressive symptoms in the NN group. Since physical activity is reduced in depressed individuals (24), it is not clear whether the absence of an exercise habit is associated with geriatric depression or whether people prone to geriatric depression simply do not like exercise. Therefore, further longitudinal studies and analyses, including the effects of genetic polymorphisms favoring exercise and the genes responsible for geriatric depression, are needed to clarify this causal relationship.

We found that exercise habits in adolescence and old age had different effects on GDS-15 scores in men and women. In men, the GDS-15 scores were significantly higher in the NN group than in the other three groups. In women, the GDS-15 scores were significantly lower in the NA and AA groups than in the NN group; however, there was no significant difference between the AN and NN groups, which was generally consistent with the results of the logistic analysis, except for the AN group in women.

The present study had some limitations. First, the participants were all older adults living in urban areas (Bunkyo-Ku, Tokyo, Japan), which may have introduced a selection bias. Education level positively correlates with cognitive function in older adults (25, 26) and can moderate the adverse effects of geriatric depression (27). In the present cohort study, participants from similar studies in Japan had a higher level of education than general older adults (11, 28, 29). Second, self-reported exercise habits may have been affected by recall bias, as participants were asked to recall their exercise habits up to 50–60 years prior. “Bukatsudo” is a traditional sports club activity commonly performed in junior and senior high schools in Japan as part of the educational curriculum (30). Students who participate in “Bukatsudo” generally engage in three to three and a half hours of daily practice 6 days a week, regardless of vacations (31). Therefore, the recall bias was likely limited. Third, this study did not account for the specific number of years participants engaged in exercise. However, it has been reported that once a student joins a club, they typically continue participating in the same activities for 3 years (30). Another potential limitation is that all study participants had at least a junior high school education, and the impact of exercise habits on mental health may differ for individuals with a lower educational level. Therefore, future studies should aim to include participants with more diverse educational backgrounds and conduct analyses that incorporate detailed information on adolescent exercise habits to better understand their long-term effects on mental health.

In conclusion, the present study showed that older adults with exercise habits in adolescence and/or old age had a lower prevalence of geriatric depressive symptoms. Thus, it is suggested that having an exercise habit in adolescence or old age may be beneficial for good mental health in old age.

## Previous presentation

The data of this study have been presented via poster previously by Huicong Shi at the 9th Asian Conference for Frailty and sarcopenia on October 28, 2023, in Singapore. The title of this talk was “Effect of exercise habits in adolescence and older age on Geriatric depression: the Bunkyo Health Study.”

## Data Availability

Some or all datasets generated and/or analyzed during the current study are not publicly available because participant consent for public data sharing was not obtained at the time of data collection; however, they can be obtained from the corresponding author upon reasonable request.

## Supplementary material

SUPPLEMENTARY FIGURE S1 Exercise participation rate (%) by ages for the non-geriatric depressive symptoms group and geriatric depressive symptoms group. (A) Men; (B) women. **p* < 0.05, significant difference between non-geriatric depressive symptoms group and geriatric depressive symptoms group, ***p* < 0.01, significant difference between non-geriatric depressive symptoms group and geriatric depressive symptoms group.

SUPPLEMENTARY FIGURE S2 Exercise participation rate (%) by four exercise groups: no exercise in either period (none-none), exercise only in adolescence (active-none), exercise only in old age (none-active), and exercise in both periods (active-active). (A) Men; (B) women. ^†^*p* < 0.05 for significant differences compared to the None-None group, *^#^p* < 0.05 for significant differences compared to the none-active group, ^§^*p* < 0.05 for significant differences compared to the active-none group for the Chi-squared tests.
