# Supplementary figures and images for: Exercise habits in adolescence and old age are positively associated with geriatric depressive symptoms: the Bunkyo Health Study
Source: Front Public Health. 2024 Nov 19;12:1405666. doi: 10.3389/fpubh.2024.1405666 (PMC11613478; doi:10.3389/fpubh.2024.1405666)

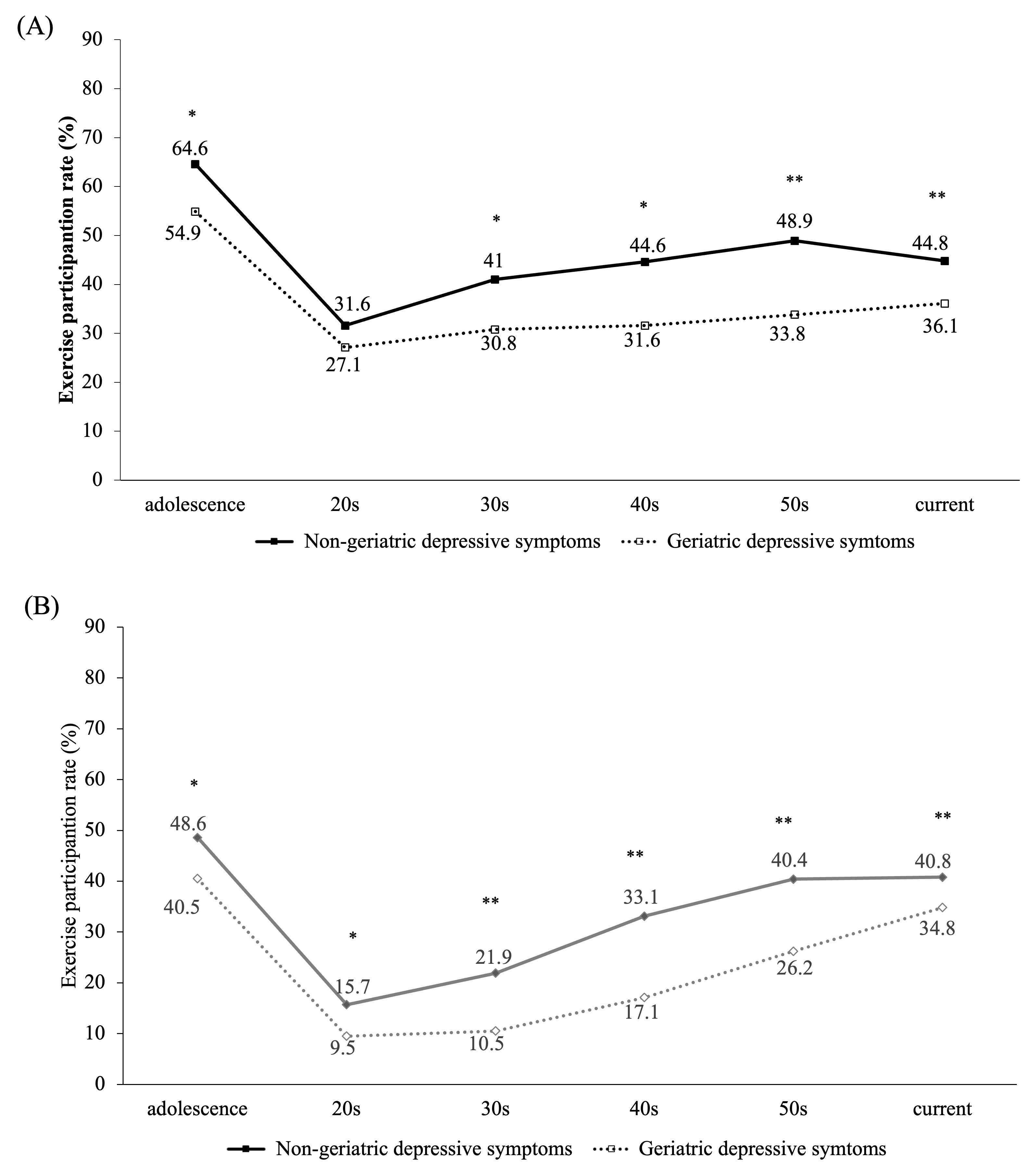

Supplement: SUPPLEMENTARY FIGURE S1 — Exercise participation rate (%) by ages for the non-geriatric depressive symptoms group and geriatric depressive symptoms group. (A) Men; (B) women. *p < 0.05, significant difference between non-geriatric depressive symptoms group and geriatric depressive symptoms group, **p < 0.01, significant difference between non-geriatric depressive symptoms group and geriatric depressive symptoms group. [file Image_1.TIF]

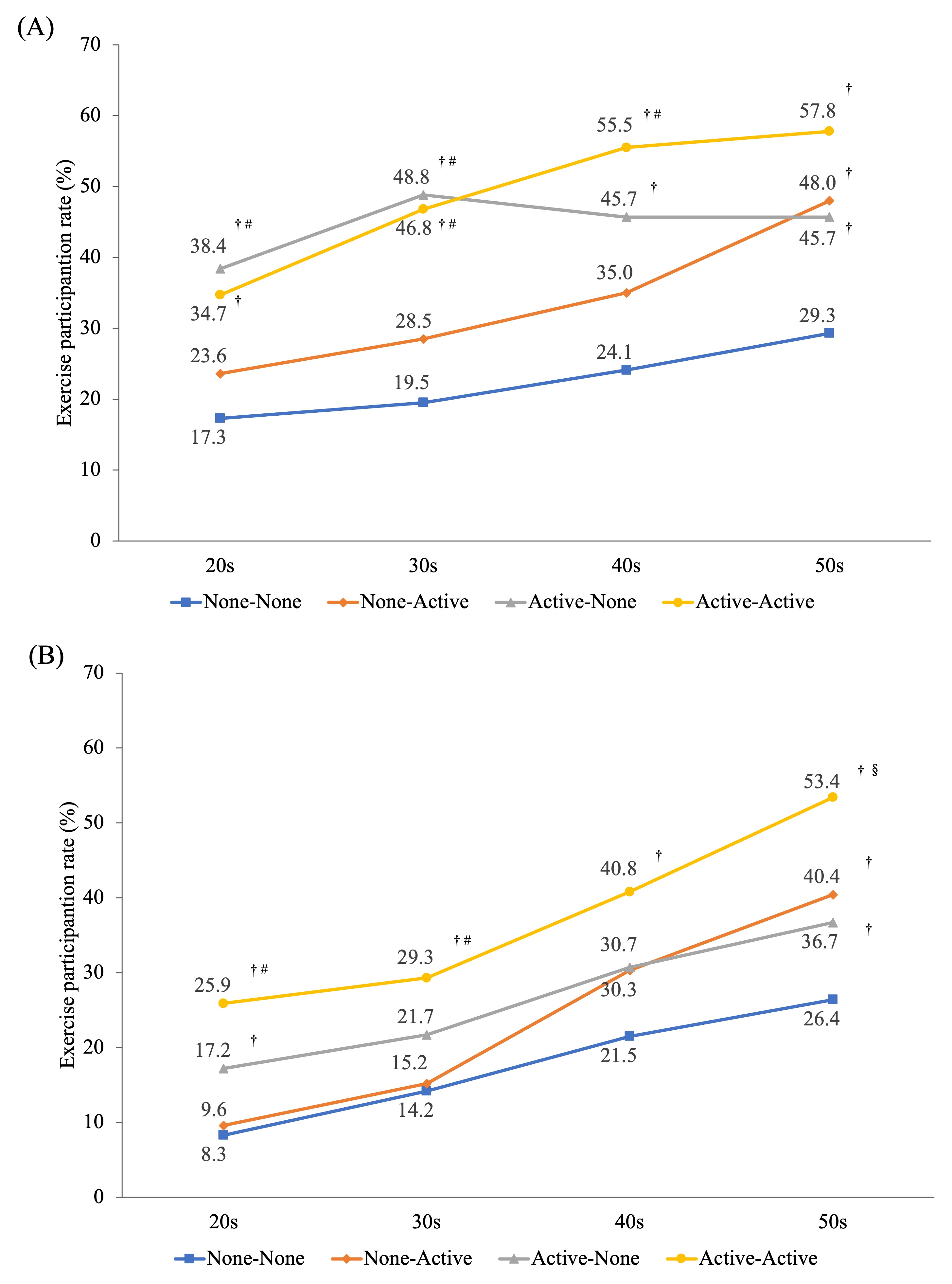

Supplement: SUPPLEMENTARY FIGURE S2 — Exercise participation rate (%) by four exercise groups: no exercise in either period (none-none), exercise only in adolescence (active-none), exercise only in old age (none-active), and exercise in both periods (active-active). (A) Men; (B) women. †p < 0.05 for significant differences compared to the None-None group, #p < 0.05 for significant differences compared to the none-active group, §p < 0.05 for significant differences compared to the active-none group for the Chi-squared tests. [file Image_2.TIF]
